# A higher far-red intensity promotes the transition to flowering in triticale grown under speed breeding conditions

**DOI:** 10.18699/vjgb-25-96

**Published:** 2025-10

**Authors:** A.O. Blinkov, V.М. Nagamova, Y.V. Minkova, N.Yu. Svistunova, S. Radzeniece, А.А. Kocheshkova, N.N. Sleptsov, А.V. Freymans, V.V. Panchenko, A.G. Chernook, G.I. Karlov, М.G. Divashuk

**Affiliations:** All-Russian Research Institute of Agricultural Biotechnology, Moscow, Russia; All-Russian Research Institute of Agricultural Biotechnology, Moscow, Russia; All-Russian Research Institute of Agricultural Biotechnology, Moscow, Russia; All-Russian Research Institute of Agricultural Biotechnology, Moscow, Russia; All-Russian Research Institute of Agricultural Biotechnology, Moscow, Russia; All-Russian Research Institute of Agricultural Biotechnology, Moscow, Russia; All-Russian Research Institute of Agricultural Biotechnology, Moscow, Russia Russian State Agrarian University – Moscow Timiryazev Agricultural Academy, Moscow, Russia; LLC “Climbiotech”, Moscow, Russia; P.P. Lukyanenko National Grain Centre, Krasnodar, Russia; All-Russian Research Institute of Agricultural Biotechnology, Moscow, Russia; All-Russian Research Institute of Agricultural Biotechnology, Moscow, Russia; All-Russian Research Institute of Agricultural Biotechnology, Moscow, Russia

**Keywords:** far-red light, red light, speed breeding, triticale, дальний красный свет, красный свет, спидбридинг, тритикале

## Abstract

It typically takes 12 to 15 years to develop a new promising variety. One of the ways to reduce this time is through speed breeding. This method allows for up to six consecutive generations of spring cereals in a single year. Although far-red light is often overlooked in speed breeding protocols, it serves as a potent inducer of accelerated flowering in various plant species. In this study, we explored the advantages of far-red light as a means to optimize the speed breeding of spring triticale. Experimental plants were cultivated under three conditions with different red to far-red ratios at 660 nm (R – red) and 730 nm (FR – far red): 1) 3.75 (R > FR); 2) 0.8 (R = FR) and 3) 0.3 (R < FR). We found that the onset of triticale flowering occurred significantly earlier at the lowest red to far-red light ratio (R/FR 0.3). On average, plants bloomed 2.6 and 4.1 days earlier in a mineral wool and a soil mixture at R/FR 0.3, respectively, than those grown at R/FR 3.75. A negative effect of higher-intensity far-red light on the reproductive system of triticale was observed. Additionally, seeds obtained from plants grown under higher-intensity far-red light showed significantly lower germination energy and capacity. No differences were found in the regenerative capacity of isolated embryos in vitro obtained from plants grown under the different spectral compositions. Our results demonstrate that the accelerated triticale development requires not only the involvement of far-red light, but also a specific red to far-red light ratio close to 0.3. A modified speed breeding protocol relying on this ratio enabled flowering to commence as early as 33.9 ± 1.2 days after sowing. The same triticale variety grown under field conditions in the Krasnodar region and in traditional laboratory growing conditions with a photoperiod of 18/6 h day/night flowered 25 to 29 days later than those cultivated under the speed breeding conditions.

## Abstract (ru)

Работа по созданию нового перспективного сорта занимает в среднем 12–15 лет. Одним из возможных решений проблемы сокращения длительности селекционного процесса становится технология спидбридинг (speed breeding). Метод, направленный на сокращение вегетационного периода, позволяет получать до шести последовательных поколений яровых злаков за один год. К сожалению, в протоколах спидбридинга уделено мало внимания дальнему красному свету – широко известному индуктору быстрого перехода к цветению. В нашей работе мы оценили возможность использования дальнего красного света для оптимизации спидбридинга яровой тритикале. Экспериментальные растения выращивали в трех вариантах освещения, различающихся соотноше- нием уровней излучения в области 660 нм (К – красный) и 730 нм (ДК – дальний красный): 1) К/ДК 3.75 (К > ДК); 2) К/ ДК 0.8 (К = ДК) и 3) К/ДК 0.3 (К < ДК). В результате установлено, что начало цветения тритикале наступало значительно раньше при самом низком соотношении красного к дальнему красному свету (К/ДК 0.3). В среднем при К/ ДК 0.3 растения, вегетирующие на минеральной вате и почвенной смеси, зацветали соответственно на 2.6 и 4.1 суток быстрее, чем при варианте К/ДК 3.75. Статистически значимой разницы по продолжительности периода от посева до цветения между вариантами К/ДК 3.75 и К/ДК 0.8 не выявлено. Показано негативное влияние увеличенной доли дальнего красного света на репродуктивную систему тритикале. У семян, сформировавшихся при К/ДК 0.3, наблюдалась значительно меньшая энергия прорастания и всхожесть. Различий в регенерационных способностях изолированных in vitro зародышей, полученных от тритикале, выросшей под светом с разным спек- тральным составом, не обнаружено. Полученные нами результаты демонстрируют, что для сокращения времени от посева до цветения тритикале важно не только наличие дальнего красного света, но и его соотношение с крас- ным, а именно использование состава, близкого к соотношению К/ДК 0.3. Модифицированный по спектральному составу света протокол спидбридинга позволил инициировать цветение уже на 33.9 ± 1.2 сутки с момента посева. Аналогичный сорт тритикале в полевых условиях Краснодарского края и классических лабораторных условиях выращивания с фотопериодом 18/6 ч день/ночь зацветал на 25–29 суток позже, чем в условиях спидбридинга.

## Introduction

Breeders and geneticists have always sought to obtain homozygous cereal lines with specified traits more rapidly, which has led to the adoption of approaches such as shuttle breeding (Mergoum et al., 2009), the production of doubled haploids (Timonova et al., 2022), the use of embryo culture (Liu et al., 2016) and molecular markers (Fedyaeva et al., 2023). However, these methods are not always accessible to specific laboratories or breeding centers, may require highly qualified personnel, and some of them do not result in the desired reduction in the time required to develop pure lines.

In recent years, speed breeding – a method based on reducing the generation time of plants to approximately two months – has been gaining popularity (Ghosh et al., 2018; Watson et al., 2018). By reducing generation time, speed breeding enables the production of up to six successive generations of spring cereals within 12 months, allowing the development of pure lines in a single year. The essence of speed breeding lies in the utilization of physical factors that reduce the time from sowing to flowering, decrease the duration of the generative stage of development, overcome post-harvest seed dormancy, and thereby minimize the time required to grow one generation. This technology is simple, low-cost, and enables work with genotypes adapted to various natural and climatic zones, enabling it to be actively integrated into diverse breeding and research programs (Hickey et al., 2017; Li et al., 2019; Vikas et al., 2021).

To reduce the time from sowing to flowering in cereals, prolonged photoperiod, a spectral composition of light including the visible light radiation range of 400–700 nm, light intensity of 450–500 μmol/(m2 · s) (Watson et al., 2018), root restriction (Zheng et al., 2023), strict temperature control (Ficht et al., 2023), elevated CO2 concentrations, and removal of tillering shoots (Tanaka et al., 2016) are employed. To shorten the maturation period, forced drying of immature seeds followed by overcoming their post-harvest dormancy (Marenkova et al., 2024) or embryo culture (Zheng et al., 2023) is used. However, there are a number of parameters, the role of which in reducing the generation time of plants is not entirely clear. One of them is the presence of far-red light during the growing period.

Far-red (FR) light (730 nm) is considered a strong inducer of photomorphogenesis and, depending on its ratio to red (R) light (660 nm), differentially affects seed germination, stem elongation, leaf blade growth, tillering, and the reduction of time from sowing to flowering (Rajcan et al., 2004; Ugarte et al., 2010; Kegge et al., 2015; Demotes-Mainard et al., 2016). Light radiation at these wavelengths and their ratio to each other (commonly described as R/FR) serve as a specific signal for plants, which is perceived by the family of phytochrome photoreceptors. In monocots, phytochromes are represented by three receptors: PhyA, PhyB, and PhyC (Demotes-Mainard et al., 2016; Kippes et al., 2020). Far-red (FR) light can exist in lower (R/FR > 1), higher (R/FR < 1), or equal (R/FR = 1) ratios relative to red light. Daylight contains approximately equal proportions of red and far-red light (1.0–1.3). This ratio decreases to around 0.6 during sunrises and sunsets. A low red-to-far-red light ratio is also observed under leaf and forest canopies, which is due to the active absorption of red light by photosynthetic pigments and the reflection of far-red light from leaves. In such cases, a low R/FR ratio serves as an indicator of the proximity of competing neighbors and triggers the shade avoidance syndrome. This syndrome manifests as enhanced elongation growth, reorientation of leaves toward regions of unattenuated daylight, and accelerated flowering, thereby improving plant survival (Demotes-Mainard et al., 2016; Smith, 2000).

In laboratory conditions, the greatest influence on the growth and development of cereals is exerted by a ratio where far-red light predominates over red light (R/FR < 1). Under light with such a spectral composition, a significant reduction in the time from sowing to flowering and a decrease in tillering shoot growth are observed (Davis, Simmons, 1994; Ugarte et al., 2010; Toyota et al., 2014; Lei et al., 2022). However, despite a number of positive opportunities for speed breeding that far-red light may provide, increasing its amount in the spectral composition of light contributes to a decrease in fertile flowers and grain number per spike (Ugarte et al., 2010; Dreccer et al., 2022).

In the protocols of speed breeding for cereal crops, the utilization of far-red light has received limited attention: in the graphs of the spectral composition of light presented in research studies, one can observe both its complete absence (Watson et al., 2018; Ficht et al., 2023) and various ratios to red light, in which the latter strongly predominates (Ghosh et al., 2018; Watson et al., 2018; Cha et al., 2022). Only in a small number of studies has far-red light been incorporated in an equal ratio with red light (Zakieh et al., 2021).

There are a number of publications on the influence of farred light on wheat (Toyota et al., 2014; Dreccer et al., 2022; Lei et al., 2024), barley (Deitzer et al., 1979; Davis, Simmons, 1994; Kegge et al., 2015), and other cereal crops (Rajcan et al., 2004; Markham et al., 2010; Huber et al., 2024). Regarding triticale, little attention has been paid to this topic, and practically no similar studies have been conducted for this crop (Kalituho et al., 1997). A similar situation exists with speed breeding studies for this crop: in open access, only a few studies can be found for spring (Cha et al., 2021) and winter (Zheng et al., 2023) triticale. Therefore, the objectives of this work are to evaluate the influence of far-red light and its ratio to red light under speed breeding conditions on the time from sowing to flowering, main agronomic traits, and reproductive system of triticale

## Materials and methods

Plant material and growing conditions. The object of the study was the spring triticale (× Triticosecale Wittm.) variety Dublet (Danko Hodowla Roślin, Poland). Dublet is one of the earliest-ripening among spring triticale varieties (Losert et al., 2016), so the obtained data can be used as an indicator of the minimum generation cycle duration under speed breeding conditions in triticale. As a doubled haploid (Arseniuk, 2019), the Dublet variety exhibits high uniformity in both the onset of developmental phases and morphological traits. Moreover, this variety is widely distributed in Europe (Lekontzeva et al., 2019; Faccini et al., 2023; Radivon, Zhukovsky, 2023) and is known to every specialist working with this crop.

The seeds treated with the fungicide Maxim (Syngenta, France) were preliminarily germinated on water-moistened filter paper in darkness at a temperature of +25 °С. After twenty-four hours, only sprouted seeds were transferred to the substrate. Trays with 110 mL cell volumes were used for cultivation. Two substrate variants were employed: 1) a soil mixture consisting of peat, chernozem, sand, and vermiculite in a ratio of 5:3:1:1 (50 g of moistened mixture per tray cell); 2) mineral wool cubes measuring 50 × 45 × 45 mm (one cube per tray cell). One sprouted seed was placed in each tray cell at 1 cm depth. The growth chamber was maintained at a constant temperature of +25–26 °С and an air humidity of 35–45 %.

For the first two weeks, plants in the soil mixture were watered as needed, and fertilization was performed once a week with Tripart fertilizer (General Hydroponics Europe, France) according to the manufacturer’s instructions. Two weeks after sowing, the plants were transferred to watering with fertilizer three times a week. Mineral wool cubes were irrigated with fertilizer daily. Foliar feeding with Siliplant (Nest-M, Russia) was performed once a week in accordance with the manufacturer’s recommendations. Treatments for diseases and pests were carried out as necessary. Tiller removal was performed during the plants growth. The photoperiod was maintained at 22/2 hours day/night according to (Watson et al., 2018). Adjustable multichromatic PWM-dimmable LED lamps (PrometheusVNIISB by Gorshkoff, Russia) were used as light sources (chip emitters: 460, 660, 735 nm, white4000K (EPIstar, China); multi-channel pulse-width modulation controller (BKD, Russia); total power of 800 watts).

As control conditions, triticale was cultivated in a Fitotron SGC 120 climatic chamber (Weiss Technik, Netherlands) with fluorescent lamps under a photoperiod of 18/6 h day/ night, a light intensity of 285 μmol/(m2 · s) at shelf level, a temperature of +22 °С, and air humidity of 65 % round the clock. Sowing and plant care were similar to those described above. As an additional control, data from long-term field trials of the P.P. Lukyanenko National Grain Center (Krasnodar region, Russia) were used. Agronomic practices and sowing dates were conventional for the region

**Fig. 1.**
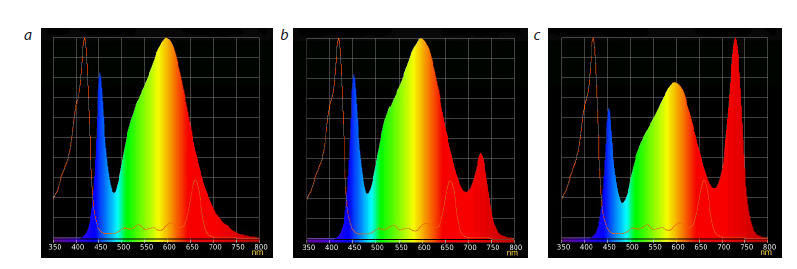
Spectral composition of light used in the experiment: a – R > FR, R/FR ratio = 3.75; b – R = FR, R/FR ratio = 0.8; с – R < FR, R/FR ratio = 0.3.

**Fig. 2.**
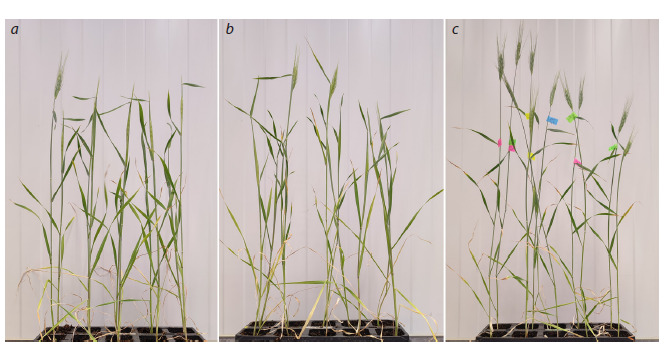
Plants on the 30th day after sowing (all plants were sown on the same day), cultivated under three lighting conditions with different spectral compositions, and at the stage of: a, b – the beginning of heading, R/FR = 3.75 (a), R/FR = 0.8 (b); c – full heading, R/FR = 0.3.

Influence of far-red light on the triticale growth stages and main agronomic traits. The degree of influence of farred light on triticale was determined by growing plants under three lighting conditions differing in the ratio of radiation levels in the 660 nm region (R – red) and 730 nm (FR – farred): 1) R/FR ratio = 3.75 (hereinafter referred to as R > FR) (Fig. 1a); 2) R/FR ratio = 0.8 (hereinafter referred to as R = FR) (Fig. 1b); 3) R/FR ratio = 0.3 (hereinafter referred to as R <FR) (Fig. 1c).

The light intensity in all variants was set to 330 μmol/ (m2 · s) at shelf level. Far-red light was introduced one week after seed germination. Lighting parameters were adjusted and verified using a PG200N spectrometer (United Power Research Technology Corp., Taiwan).

The onset of growing stages was assessed individually for each plant according to (Zadoks et al., 1974). The onset of the heading stage was defined as the day when the spike fully emerged from the flag leaf sheath (phase Z5.9). The onset of the flowering stage was defined as the day when the first anthers appeared on the spikes (phase Z6.1).

To evaluate the influence of far-red light on triticale, an analysis of the main agronomic traits of all experimental plants was conducted based on the following parameters: plant height (cm), spike length (cm), vegetative weight of the dried spike and culm (g), number of spikelets (pcs.) and grains (pcs.) per spike, number of grains per spikelet (pcs.), and weight of 1,000 grains (g).

Effect of far-red light on seed viability. The evaluation of the influence of far-red light on seed viability indicators was conducted using two methods: 1) by culturing immature embryos; and 2) by germinating seeds on filter paper. In the first method, embryo isolation was performed on the 15th day after flowering. Caryopses were sterilized in a 50 % solution of the commercial agent “Belizna”, followed by three washes with sterile distilled water. Embryo isolation was carried out under an Olympus SZ61 stereoscopic microscope (Olympus, Japan). Cultivation was performed in Petri dishes containing agar-solidified Murashige and Skoog medium (Murashige, Skoog, 1962). The cultivation lasted for 10 days under a photoperiod of 22/2 h day/night, a light intensity of 80 μmol/ (m2 · s), and a temperature of +24 °С.

In the second method, starting from the 17th day after flowering, the amount of watering was gradually reduced until it was completely discontinued on the day of spike cutting, which occurred on the 20th day after flowering. The cut spikes were placed in paper bags, which were subjected to forced drying at a temperature of +28 °С for 7–10 days, depending on the drying rate. After drying, the spikes were threshed, and the seeds were stored in paper bags at room temperature for one week. Next, the seeds were placed in Petri dishes on filter paper moistened with a 0.5 mg/L solution of gibberellic acid (Sigma-Aldrich, USA) and incubated under cold pretreatment conditions (+4 °С, darkness, three days), followed by germination in darkness at +25 °С. Germination energy was assessed on the third day, and germination capacity was evaluated on the seventh day after placing the Petri dishes with seeds at +25 °С.

Statistical analysis. To evaluate the degree of influence of the spectral composition of light on the vegetative period of triticale, a twofold replication was used for each variant, with 10 plants in each replication. In total, 120 plants were analyzed. The number of days from sowing to flowering of each individual plant was assessed. Under field conditions, the number of days from sowing to mass flowering was evaluated.

To assess the regeneration capacity and viability of isolated embryos, a fourfold replication was employed, with 10 isolated embryos in each replication. The evaluation of germination energy and seed germination capacity was performed in fourfold replication. Each replication contained 50 seeds.

Statistical processing was performed using the R programming language (version 4.3.2). The influence of the spectral composition of light on various indicators of triticale plants was assessed using one-factor analysis of variance (ANOVA), followed by multiple comparisons of mean values using Tukey’s test to determine significant differences between plant groups.

## Results

**Effect of far-red light on triticale growth stages**

As a result of the conducted experiment, the one-factor analysis of variance revealed a statistically significant reduction in the time from sowing to the onset of flowering in plants grown under the light with the spectral composition of R/ FR = 0.3 compared to other lighting variants ( p < 0.05). This trend was observed in both substrate variants. Plants under the light with the spectral composition of R/FR = 0.3 flowered on average 2.6 and 4.1 days faster when using mineral wool and soil mixture, respectively, than those under the light with the spectral composition of R/FR = 3.75. No statistically significant difference in the duration of the period from sowing to flowering was found between the R/FR = 3.75 and R/FR = 0.8 variants ( p > 0.05) (Table 1).

**Table 1.**
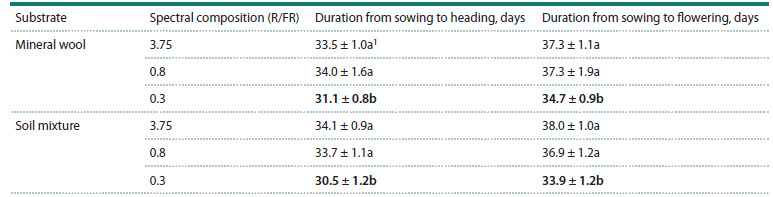
Mean values ± 95 % confidence interval for heading and flowering dates in plants of the Dublet variety grown under three lighting conditions with different spectral compositions Notе. 1 Values followed by the same letter do not differ significantly (p > 0.05) according to Tukey’s test. Bold type indicates values that are significantly different from the other variants (p < 0.05).

**Effect of far-red light on main agronomic traits of triticale**

No significant differences in vegetative weight or straw height were observed among triticale plants grown under light with different spectral compositions ( p > 0.05). A significant influence of the high amount of far-red light on spike productivity was evident (Table 2). When triticale was cultivated under R/FR = 0.3, plants on both substrate variants formed shorter spikes with fewer spikelets, leading to a reduction in the vegetative weight of the spike and the number of grains per spike ( p < 0.05). An increased amount of far-red light resulted in fewer grains per spikelet, but only in plants grown on the soil mixture. Despite this, a statistically significant increase in 1,000-grain weight was observed in plants grown under R/ FR = 0.3 on both substrate variants ( p <0.05). In the majority of cases, no statistically significant difference was detected between the R/FR = 3.75 and R/FR = 0.8 variants in terms of productivity indicators

**Table 2.**
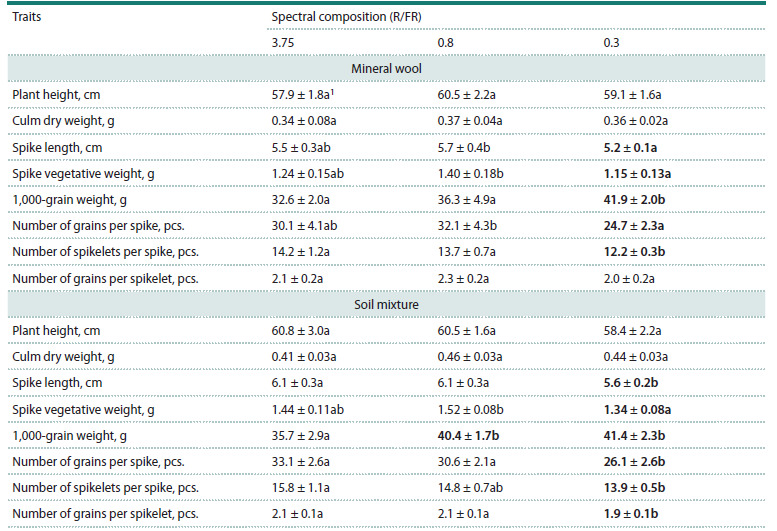
Mean values ± 95 % confidence interval for the main agronomic traits of the Dublet variety grown under three lighting conditions with different spectral compositions Notе. 1 Values followed by the same letter do not differ significantly (p > 0.05) according to Tukey’s test. Bold type indicates values that are significantly different from the other variants (p < 0.05).

**Effect of far-red light on seed viability and germination**

A statistically significant decrease in germination energy and capacity was observed in seeds obtained from plants grown under an increased amount of far-red light ( p < 0.05). In isolated embryos in vitro derived from plants cultivated under light with different spectral compositions, no statistically significant differences in regeneration frequency were detected ( p> 0.05) (Table 3). Already on the third day after the start of cultivation, regardless of the lighting conditions of the donor plants, the embryos developed coleoptiles and roots, and by the tenth day of cultivation, the embryos exhibited one fully formed leaf and a well-developed root system

**Table 3.**
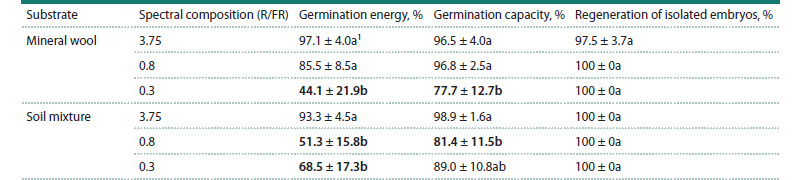
Germination energy and capacity, as well as regeneration frequency of isolated embryos obtained from plants grown under three lighting conditions with different spectral compositions Notе. 1 Values followed by the same letter do not differ significantly (p > 0.05) according to Tukey’s test. Bold type indicates values that are significantly different from the other variants (p <0.05).

**Control plants growing**

Plants under all control conditions exhibited a prolonged germination– flowering period. According to long-term cultivation data in the Krasnodar region, the anthesis of triticale variety Dublet occurred on days 60–64 when sown in early March and on days 50–52 when sown in early April. Under the conditions of a climate-controlled chamber with a photoperiod of 18/6 h day/night, triticale reached the flowering stage 62.5 ± 2.0 and 59.2 ± 2.6 days after sowing on mineral wool and on soil mixture, respectively.

## Discussion

Speed breeding has demonstrated its popularity across various fields of genetics, breeding, and biotechnology (Ghosh et al., 2018). Modifications of established protocols are being implemented, including simplifying their organization, transitioning to high-throughput capacity, incorporating molecular genetics methods, and integrating them into the breeding process (Kigoni et al., 2023; Marenkova et al., 2024). At present, speed breeding protocols have been successfully tested in numerous cereal species (Watson et al., 2018; Cha et al., 2021). Despite active work in this area, most published studies on cereal speed breeding have not adequately addressed one of the strongest inducers of shortening the sowing-to-flowering period – far-red light. However, under speed breeding conditions, its efficacy has been demonstrated for crops such as rapeseed (Song et al., 2022), amaranth (Jähne et al., 2020), and pepper (Choi et al., 2023).

The shortening of the vegetative period is one of the primary manifestations of shade avoidance syndrome, initiated by far-red light in the photoperiodic regulation of flowering. Light with an increased amount of far-red light is perceived by leaves and activates phytochrome photoreceptors, primarily PhyA and PhyB. Phytochromes trigger the expression of the central flowering regulator gene CONSTANT (CO), which, in turn, induces FLOWERING LOCUS T (FT) – the florigen in the vascular bundles of leaves. The FT protein moves from the leaves to the shoot apical meristem and, together with the FD protein (product of the FLOWERING LOCUS D gene (FD)), initiates the activity of genes such as SUPPRESSOR OF OVEREXPRESSION OF CO1 (SOC1) and APETALA1 (AP1), which determine the development of floral meristems (Demotes-Mainard et al., 2016; Sheerin, Hiltbrunner, 2017; Lebedeva et al., 2020).

To evaluate the effect of far-red light under speed breeding conditions, we conducted an experiment involving the cultivation of spring triticale on two types of substrates and under three lighting variants differing in spectral composition, characterized by varying ratios of red to far-red light.

The experiments conducted by us demonstrated a significant influence of an increased amount of far-red light (R < FR, R/FR = 0.3) on the onset of the flowering phase in triticale. Plants exposed to the light with the spectral composition of R/FR = 0.3 flowered 33.9 ± 1.2 and 34.7 ± 0.9 days after sowing when grown on soil mixture and mineral wool cubes, respectively, which is 4.1 and 2.6 days faster than under the light with R/FR = 3.75. No statistically significant difference in the duration of the vegetative period of triticale was observed under light spectra with R > FR and R = FR. The obtained results indicate that in order to shorten the sowing-to-flowering period in triticale, not only the presence of far-red light but also its ratio to red light is critical, specifically the use of a composition close to R/FR = 0.3 (Fig. 2). Our findings align with those of several other studies reporting similar results in cereals (Deitzer et al., 1979; Davis, Simmons, 1994; Toyota et al., 2014).

Despite the difference in flowering onset timing, amounting to 2.6 and 4.1 days, far-red light can be considered a valuable addition for creating conditions aimed at shortening the vegetative period of plants. This is because if the sowing–flowering period can be reduced by 3–4 days in one generation, the cumulative effect when sequentially growing six generations (a number that typically facilitates the production of a pure line) could reach up to 20 days.

Currently, only a limited number of studies are dedicated to speed breeding of spring and winter triticale, demonstrating the high responsiveness of this crop to factors influencing the shortening of the vegetative period (Cha et al., 2021; Zheng et al., 2023). It has been shown that for spring triticale, the average time from sowing to heading ranges between 33–42 days depending on the genotype (Cha et al., 2021), whereas spring bread wheat under speed breeding conditions flowers, depending on the genotype, between 35.7 and 75 days after sowing (Ghosh et al., 2018; Watson et al., 2018; Cha et al., 2020). Our work confirms the significant impact of the speed breeding method on reducing the vegetative period in triticale: the spectrally modified protocol enabled the initiation of flowering as early as 33.9 ± 1.2 days after sowing. The same triticale cultivar under field conditions in the Krasnodar region and classical laboratory cultivation conditions flowered 25–29 days later than under speed breeding conditions.

The results of the evaluation of triticale yield structure did not reveal significant changes in the height of the studied plants when grown under the light with a spectral composition containing an increased amount of far-red light, although numerous studies report stem elongation in cereals under far-red light (Kegge et al., 2015; Lei et al., 2022). Shade avoidance syndrome, which causes shoot elongation, has also been absent in other studies where far-red light was used as a supplement to shorter wavelengths (400–680 nm) (Huber et al., 2024). This suggests that the use of increased amounts of far-red light in the optical radiation spectrum under triticale speed breeding conditions does not lead to such an inconvenient factor in practice as the formation of tall plants and their lodging.

The data obtained by us demonstrated a strong influence of far-red light on the productivity of triticale spikes. In plants of the Dublet variety grown under the light with the spectral composition of R/FR = 0.3, a shorter spike with fewer spikelets was formed, leading to a significant reduction in the vegetative mass of the spike and the number of grains per spike. Similar results have been reported in bread wheat, where an increased amount of far-red light reduces the number of fertile flowers and the number of grains per spike (Ugarte et al., 2010; Dreccer et al., 2022), which is likely associated with the inhibitory effect of far-red light on plant nitrogen assimilation (Lei et al., 2024).

Despite the negative influence of far-red light on spike productivity components, the 1,000-grain weight of plants grown under an increased amount of far-red light in the spectral compositions was significantly higher. This may be associated with the Emerson effect, which involves enhanced photosynthetic efficiency when far-red light is used in combination with shorter wavelengths (400–680 nm) (Huber et al., 2024).

Additionally, during our study, a statistically significant negative impact of far-red light on germination energy and capacity was detected. The germination capacity of seeds obtained from plants grown under the light with the spectral composition of R/FR = 0.3 ranged from 77.7 ± 12.7 to 89.0 ± 10.8 % depending on the growth substrate. At the same time, the regeneration frequency of isolated embryos in vitro was equally high for all seeds, regardless of the lighting conditions under which the donor plants were cultivated. Given that in cereals, the speed breeding system is compatible with the single-seed descent method (Alahmad et al., 2018; Watson et al., 2018), where one seed per spike is selected for each subsequent generation to preserve genetic diversity and prevent the expansion of cultivation areas, the use of high amounts of far-red light in the spectral compositions will not become a limiting factor when cultivating plants using this method. However, it should be noted that when the primary goal of plant cultivation is propagation and obtaining seeds with good germination capacity, it is necessary to reduce the amounts of far-red light in the optical radiation spectrum to a level where R/FR > 1.

## Conclusion

Our study demonstrated that under speed breeding conditions, the use of the highest amount of far-red light in the spectral composition (R/FR = 0.3), compared to the spectrum where the R/FR ratio is 3.75, resulted in a statistically significant reduction in time from sowing to flowering by 2.6 and 4.1 days for plants grown in mineral wool and soil mixture, respectively. No statistically significant difference in the duration from sowing to flowering was detected between the R/FR = 3.75 and R/ FR = 0.8 variants. The speed breeding protocol with a modified light spectrum induced flowering as early as 33.9 ± 1.2 days after sowing. The same triticale variety flowered 25–29 days later under field conditions in the Krasnodar region and conventional laboratory cultivation with a photoperiod of 18/6 h day/night compared to modified speed breeding conditions. No statistically significant increase in plant height was observed when using the highest amount of far-red light in the spectral composition. A negative influence of far-red light on spike parameters (length, vegetative weight, number of spikelets and grains per spike) as well as germination energy and capacity was detected. It can be reasonably assumed that increasing the amount of far-red light in the optical radiation spectrum (R/FR = 0.3) could serve as a beneficial addition to speed breeding conditions not only for triticale but also for other cereals.

## Conflict of interest

The authors declare no conflict of interest.
